# A Novel Charging Method for Underwater Batteryless Sensor Node Networks

**DOI:** 10.3390/s21020557

**Published:** 2021-01-14

**Authors:** Judith Santana Abril, Graciela Santana Sosa, Javier Sosa, Tomas Bautista, Juan A. Montiel-Nelson

**Affiliations:** Institute for Applied Microelectronics (IUMA), University of Las Palmas de Gran Canaria, 35015 Las Palmas de Gran Canaria, Spain; jsabril@iuma.ulpgc.es (J.S.A.); gsantana@iuma.ulpgc.es (G.S.S.); bautista@iuma.ulpgc.es (T.B.); montiel@iuma.ulpgc.es (J.A.M.-N.)

**Keywords:** wireless charging, wireless power transfer (WPT), batteryless sensor node, wireless sensor network (WSN), precision aquaculture, offshore fish farm

## Abstract

In this paper, we present a novel charging method for underwater batteryless sensor node networks. The target application is a practical underwater sensor network for oceanic fish farms. The underwater sections of the network use a wireless power transfer system based on the ISO 11784/11785 HDX standard for supplying energy to the batteryless sensor nodes. Each sensor has an accumulator capacitor, which is charged for voltage supplying to the sensor node. A new distributed charging scheme is proposed and discussed in detail to reduce the required time to charge all sensor nodes of the underwater sections. One important key is its decentralized control of the charging process. The proposal is based on the self disconnection ability of each sensor node from the charging network. The second important key is that the hardware implementation of this new feature is quite simple and only requires to include a minimal circuitry in parallel to the current sensor node antenna while the rest of the sensor network remains unaltered. The proposed charging scheme is evaluated using real corner cases from practical oceanic fish farms sensor networks. The results from experiments demonstrate that it is possible to charge up to 10 sensor nodes which is the double charging capability than previous research presented. In the same conditions as the approach found in the literature, it represents reaching an ocean depth of 60 m. In terms of energy, in case of an underwater network with 5 sensors to reach 30 m deep, the proposed charging scheme requires only a 25% of the power required using the traditional approach.

## 1. Introduction

Nowadays, wireless power transfer (WPT) technologies have enabled the emergence of several battery or batteryless based applications. Depending on the energy transfer mechanism used in the WPT, these are classified as far or near field. Far-field based solutions use electromagnetic transmission to high frequencies. On the other hand, near-field based solutions use magnetic transmission and low frequencies. Near or far approaches are used depending on the application. In both cases the design and optimization of the antenna elements or the charging circuitry in terms of its efficiency define a common research area [1] on all WPT literature.

Removing tedious power wires and their connectors on battery–based systems simplifies their use. This is the case, for example, of the new consumer electronics like activity trackers, smart-watches or mobile phones where a WPT charging system makes it possible to remove the power connector and therefore to improve the water insulation of the entire housing [2].

With mobility policies to reduce greenhouse gas emissions that are based on the use of Electric Vehicles (EVs), where the development of vehicle battery charging systems has become a hot topic in the research area of WPT technologies [3,4,5]. In a similar way, the unmanned underwater vehicles (UUV) are mainly powered by batteries and WPT is also a hot research topic [6].

Furthermore, the WPT technologies have promoted the rising of new medical applications where traditional wired solutions are not efficient or impossible to apply [7,8]. It is well known that the use of wired solutions in medical electronic devices increases the appearance of infections and diseases related to the location of the implanted device or its connector [9].

In addition, the WPT technologies have to be proven useful in the field of wireless sensor networks (WSN) [10]. The existing literature covers a wide spectrum of solutions based on WSN and WPT technologies. For example, the use of radio frequency identification (RFID) technologies allows the use of batteryless sensor nodes [11].

In [12], the authors present a low–cost electronic tagging system for bee monitoring. The animals are uniquely identified with a batteryless tag. In this case the tag allows one to determine its behavior in terms of several parameters including the number of times and duration of their visit to a feeding station or honeycomb. Another example application in WSNs is the presented in [13], where the authors present the design and implementation of a wireless sensor node architecture based on RFID devices to monitor the manufacturing process of Sardinian Carasau bread.

A mixed wired and wireless sensor network solution is also presented in [14]. The approach is a sensors node network deployed in offshore fish farm cages based on wired connections where several wireless interconnections are inserted using a WPT method to minimize the effect of short circuits in the power distribution.

From the point of view of the WPT technologies, the literature studies are focused in three mainly areas: topologies, devices and control strategies. By definition a wireless sensors network is composed of multiple sensor nodes. This scenario defines a multi–receiver environment [15]. For example, in [16], the impedance matching for simultaneous WPT devices is analyzed. In other words, the classical structures in series and in parallel of WPT to determine their main characteristics and usability in load systems are studied [2].

The optimal load to maximize the efficiency of a single input multiple output WPT (SIMO–WPT) system is presented in [17]. The objective of the authors requires that the load of the systems to charge varies depending on the total number of systems present in the charging area. In [18] is presented a multiple input multiple output WPT (MIMO–WPT) system for batteryless wireless sensors. Here the key point it is to maximize the energy received in the sensor node reducing the dependency of the energy transmitter.

In [19] authors present a control strategy and propose a circuit for a bidirectional wireless charging system oriented to a sensors network based on Unmanned Aerial Vehicles (UAVs). Depending on the scenario, each UAV can charge its batteries from a ground charging base or act as an aerial charging base to other UAVs. An approach to minimize the cross-coupling effect between multiple systems under charging is presented in [20]. Authors use a time–sharing strategy based on a centralized control system connected to every charged element.

Most of the literature research concludes that the presence of multiple sensor nodes/ charged systems must be taken into consideration due to its influence along the WPT process [14,18,20,21,22].

The key contributions of this proposal are the following:

- A novel distributed charging method is presented and discussed in detail including practical considerations for a real batteryless sensor node network application.
- The optimization of the charging process is performed maximizing the number of total fully charged sensor nodes and decreasing the required time for the complete set of sensor nodes and the deepest one in the network.
- The proposal implementation requires only the addition in parallel to each sensor node antenna a reduced set of commercial components and the rest of the network remains unaltered.

The rest of this paper is organized as follows. In Section 2, the target application, which is to monitor an oceanic offshore fish farm, is introduced. In other words, the underwater sensor network is presented. In Section 3, first the sensor node is presented. Then its design considerations and the disconnection idea are discussed. In addition, in this section a circuit modification is proposed. Next, experiments and results are evaluated in Section 4. Finally, Section 5 gives the conclusions of this work.

## 2. Underwater Sensor Network

**Figure 1:**
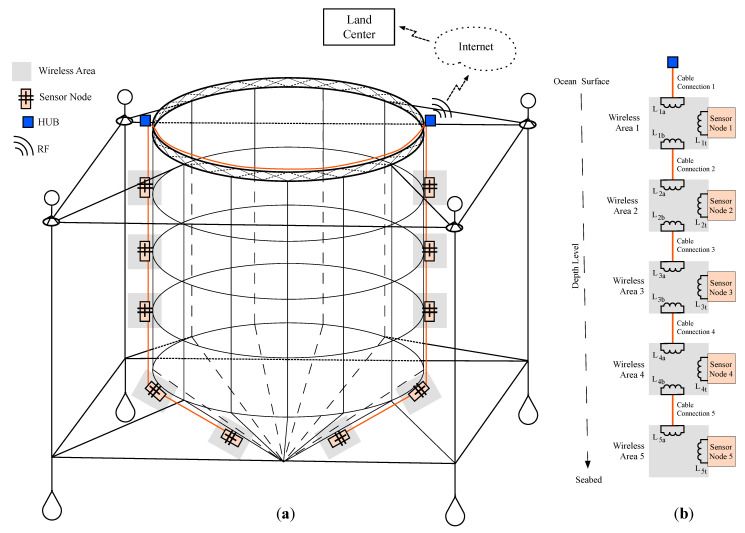
Example of underwater sensor network: (**a**) sensor network deployment in an offshore cage and (**b**) details of a network branch with five sensor nodes.

Based on the proposal presented in [14], Figure 1a shows an example of the deployment of an underwater sensor network in an offshore oceanic cage [23]. The control and monitor center located in land is connected to the electronic infrastructure of the offshore cage through Internet using any common RF technology like 3G, 4G, WiMAX or WiFi. The choice of communication technology is made according to the distance between the marine cage and the on land center. In the cage, there is a main hub that concentrates all the communications between the on land center and the sensor network. The sensor network is made up of several network branches. All network branches are connected to the cage hub. The hub is always placed at the ocean surface level.

The network branch follows a modular philosophy. Figure 1b shows a network branch example with five modules (N=5). Each module defines a wireless area *i*, where i∈[1,5]. This wireless area includes a transmission antenna $L_{ia}$ and a reception antenna $L_{ib}$. The transmission antenna $L_{ia}$ collects energy from the previous wireless area i−1. The reception antenna $L_{ib}$ provides energy to the next wireless area i+1. Obviously, the deepest wireless area of the network branch has no reception antenna. In addition, each wireless area includes a sensor node. The connection between modules are done using a cable. That is, the connection between a transmission antenna of a wireless area and the reception antenna of the previous wireless area is made using traditional wires. Finally, the transmission antenna of the node close to the ocean surface is connected to a sinusoidal generator located in the cage hub.

### 2.1. Network Branch Optimization

**Figure 2:**
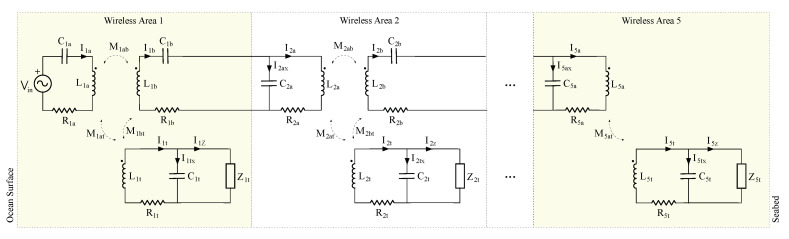
Wireless areas network branch circuital model for five sensor nodes.

Figure 2 presents the circuital model for the network branch shown in Figure 1b. As it was previously introduced, this circuit is divided by wireless areas. They are enumerated and identified with correlative numbers which are increased with the depth of the node. The first wireless area is physically located close the surface level of the ocean. This first wireless area includes a low frequency RFID sinusoidal source $V_{in}$, a transmission antenna $L_{1a}$ and its tunning capacitor $C_{1a}$. Each wireless area is designed to have at least one sensor node. The energy in the sensor node is received by antenna $L_{t}$ which has its own tunning capacitor $C_{t}$. If there is a sensor node deeper than the current one, the wireless area includes a transmission antenna $L_{b}$ and its tunning capacitor $C_{b}$ connecting the current wireless area with the deeper one.

Following the authors optimal formulas proposed in [14], we set the values for those components of a network branch with five sensor nodes. In this setup, we also assume that the maximum distance between the sensor nodes and the hub is 6 m. Table 1 presents the optimal computed values. The use of those values guarantees the optimal transmission of the energy in a network branch of 30 m long.

### 2.2. Sensor Node Charging Process

**Figure 3:**
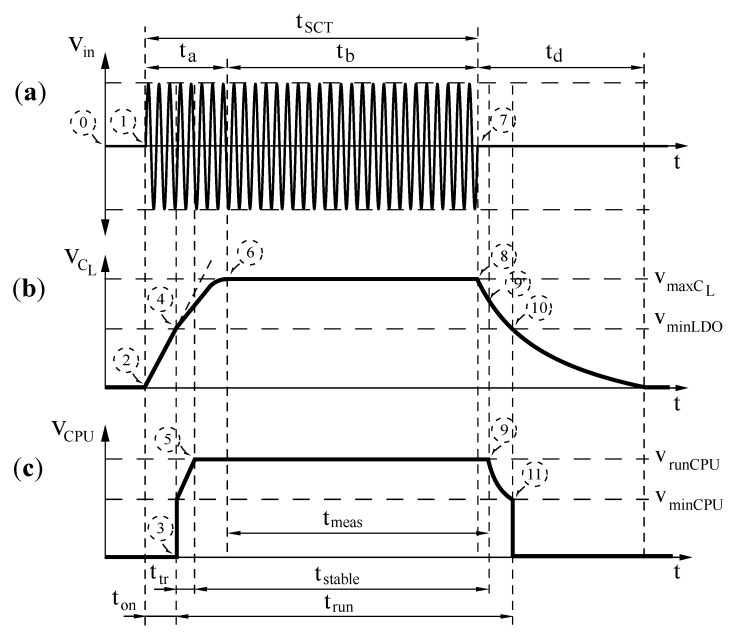
ISO 11784/5 HDX usage to charge a batteryless sensor node: (**a**) the sinusoidal charging tone SCT, (**b**) the energy accumulator capacitor $C_{L}$ and (**c**) the voltage of the sensor node CPU.

All sensor nodes used in this research are batteryless. In addition, they are compliant with the ISO 11784/11785 HDX standard. Figure 3 depicts the charging procedure. As shown in Figure 3a, the ISO 11784/11785 standard considers three time periods. Two are while the excitation signal is present and the other is specified immediately after finishing the excitation signal. The first one is the charging time $t_{a}$. In this period, the sensor node accumulates the received energy. Once the charging time is over, despite the presence of the excitation signal, the sensor node cannot store more energy since it is fully charged. We named this remaining time as $t_{b}$. Therefore, the SCT time $t_{SCT}$ is equal to $t_{a}+t_{b}$. Finally, the last time period begins when the excitation signal disappears. The energy accumulated is used to execute several tasks in the sensor node. We named this time as discharging time $t_{d}$.

In the worst case scenario, the batteryless sensor node is without stored energy (see label 0 in Figure 3a). Obviously, the voltage in its energy accumulator capacitor is zero ($V_{C_{L}}=0$). Therefore, the sensor node CPU is completely turned off. The charging process begins when the sensor node receives a SCT of 134.2 kHz as shown on label 1 in Figure 3a. As a consequence, the voltage of the energy accumulator $C_{L}$ increases its value. Due to safety requirements, the $V_{C_{L}}$ is limited to a maximum charging voltage. This behavior is observed on labels 2, 4 and 6 of Figure 3b along the time $t_{a}$.

A sensor node includes a smart circuitry providing the minimum intelligence to control the charging and measurement process. It can be a simple digital circuit implemented using an application-specific integrated circuit (ASIC) or a complex system on a chip (SoC) including a microcontroller. In both cases, it is mandatory to convert the energy received and stored in the sensor node to the power supply voltage range required by those digital circuits. Thus, a low–dropout (LDO) regulator is connected directly to the energy accumulator capacitor $C_{L}$.

In a similar way, the regulator requires an input voltage range to work correctly. The output terminal of the regulated voltage is activated only when its input terminal voltage is above $V_{minLDO}$. So, during the charging of the energy accumulator capacitor, the smart circuit of the sensor node is turned on at $t_{on}$ (see the label 3 in Figure 3c). Then, the equivalent load over the charging process is increased. This effect is observed in label 4 of Figure 3b with the charging slope of $V_{C_{L}}$.

Then, the smart circuit supply voltage increases its value from $V_{minCPU}$ to $V_{runCPU}$ as shown in Figure 3c. In this elapsed time $t_{tr}$, despite the smart circuit is working, we cannot execute measurements due to voltage variations (see labels 3 and 5). However, it is possible, for example, to set the configuration of the measurement devices or execute some data integrity checking and/or compression.

We need to take into consideration that to execute a measurement increases the energy consumption. Therefore, the fully charged energy accumulator capacitor will require extra time when a measurement is active along the charging process. This scenario produces a critical failure, especially the less power availability that the charging tone has.

As soon as the charging tone disappears (see label 7 in Figure 3a), the sensor node uses the energy stored in its energy accumulator capacitor to keep the smart circuit and the measurement running correctly as shown in label 8 of Figure 3b. There exists a non-return point where the remaining energy at the sensor node accumulator is not able to keep supply voltage stable. At this point, which is labeled as step 9, the LDO reduces the CPU supply voltage until it reaches the minimum input voltage to disconnect its regulation (see labels 10 and 11).

Given a sensor node *i* from a network branch, we define its charging time $t_{a_{i}}$ as the required time to charge its energy accumulator capacitor. In addition, its fully charging time $t_{b_{i}}$ is defined as the time which the accumulator capacitor remains with the maximum voltage $V_{maxC_{L}}$ with the presence of the SCT. Finally, the discharging time $t_{d_{i}}$ is the time required to reach a zero voltage at the accumulator capacitor after finishing the SCT.

From the point of view of a smart circuit for a given node *i*, we define its turn on time $t_{on}$ as the time required to reach its operating voltage once a charging tone is present. In addition, $t_{run}$ is the time where the power supply regulator applies a voltage to the smart circuit allowing the execution of auxiliary and/or measurement tasks. It is possible to identify a $t_{stable}$ which it is defined as the time where the power supply voltage is set to $V_{runCPU}$. Finally, we define the measurement time $t_{meas}$ as the period within $t_{stable}$ which allows to execute a measurement process.

## 3. Sensor Node Optimization

### 3.1. Sensor Node Circuit

**Figure 4:**
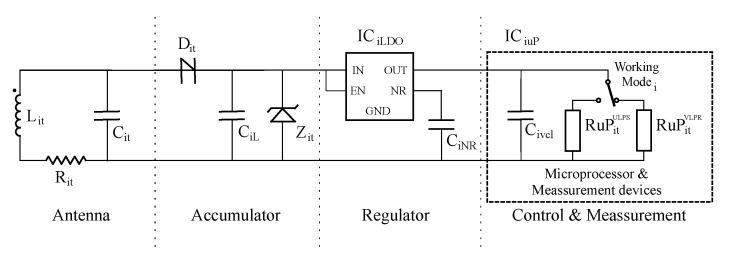
Proposed batteryless sensor node equivalent circuit.

In this research, we use as sensor node model the circuit presented in Figure 4. It is made with commercial off-the-shelf (COTS) devices. Basically, it is divided into four independent stages. The first one is the antenna stage. It contains a coil $L_{it}$ that works as a reception antenna. In addition, a compensation capacitor $C_{it}$ is connected using a parallel configuration. The antenna parasitic resistance $R_{it}$ is also taken in consideration as shown in the diagram.

The second stage is the accumulator. It includes a half wave rectifier $D_{it}$, an energy accumulator capacitor $C_{iL}$ and a voltage limiter $Z_{it}$. The voltage regulator is added to provide the power supply to the smart circuit. In this sensor node, our smart circuit is a SoC which includes a microprocessor and the measurement devices.

Finally, we model the SoC behavior with an equivalent capacitor and two resistors named $C_{ivcl}$, $RuP_{it}^{UPLS}$ and $RuP_{it}^{VPLR}$, respectively. Each equivalent resistor corresponds to a working state power consumption of the SoC. One is the ultra low power stop (ULPS) mode, where it is consuming the minimum energy waiting for a wake-up event. In this state, all the measurement devices are powered off. The other resistor models the power consumption of the SoC in very low power run (VLPR) mode. In addition, this last resistor includes the consumption of the measurement devices executing experiments. Table 2 presents the values of the components used in our experiments.

In order to obtain the timings using the ISO 11784/5 HDX standard, we also need to determine the basic parameters presented in Table 3. Based on power consumption and computation capabilities, we choose a MKL17Z256 from NXP as microprocessor for our sensor node. It is designed to run in the 1.71 V and 3.6 V power supply range. We have chosen a 2.7 V voltage since it is the highest power supply voltage that allows one to use the VLPR mode. We require the highest voltage possible to maximize the operating range of the analog to digital converters (ADCs) used for measurement purposes.

Once is selected the LDO regulator with a 2.7 V as output voltage, the next step is to limit its maximum input voltage. For a TPS71727, this value is 6 V. Taking into account the operation of the LDO, it activates its output at 2.25 V when the input reaches 2.45 V. Finally, other important temporal limits are defined by the ISO 11784/5 standard where it is set the minimum, typical and maximum duration of the SCT.

**Figure 5:**
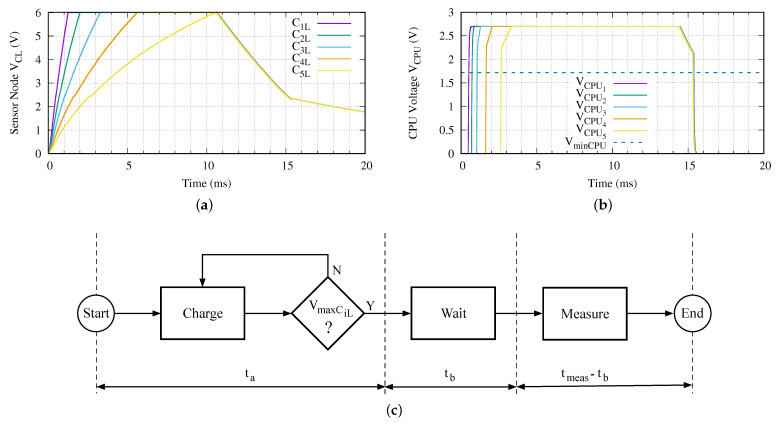
Charging behavior of all sensor nodes in a five levels (N=5) network branch: (**a**) energy accumulator capacitor voltage, (**b**) SoC power supply voltage and (**c**) executed algorithm in the sensor node.

Given a network branch like the one proposed in Figure 2, with all sensor nodes equal to the presented in Figure 4 and using the ISO 11784/5 HDX, Figure 5 depicts the charging procedure of all five sensor nodes and the power supply applied to each microprocessor.

In order to follow this standard, each sensor node implements the algorithm shown in the block diagram presented in Figure 5c. Obviously, the first step is to charge the energy accumulator. Once the SoC is powered, it goes into ULPS state. Before stopping, we program a wake-up interruption which turns on the SoC when the $V_{maxCL}$ is reached in $C_{CL}$. As soon as this charging time $t_{a}$ is achieved, the SoC is programmed using its low power timer (LPTMR) to wait the charging of the deepest sensor node. Along this waiting time, the SoC is in ULPS mode. After the wake-up of the LPTMR interruption, the measurement/experiment is executed in VLPR mode.

The duration of the SCT, in this experiment, is equal to the charging time of the deepest sensor node. That is:

(1) $$t_{SCT}=t_{5a}=t_{ia}+t_{ib}=10.58\;\mathrm{ms},\;\forall i\in \left[1,5\right]$$

In this experiment, the maximum measurement time is 3.9 ms. Increasing this time is done in two ways. The first one implies to keep the SCT active for longer. This solution is applied if the extended charging time contributes to the required extra energy. If this soft method is not viable, the value of $C_{iL}$ must be increased. However, the consequences of this hardware solution implies recomputing all the values for the time parameters for network branch.

### 3.2. Design Considerations Discussion

This network branch circuit is a single input multiple output wireless power transfer (SIMO–WPT) system. That is, there exists only a single source of energy and there are several consumers [15]. In SIMO–WPT systems, one key problem is the cross-coupling between the elements to charge. In the scenario of the current application, due to the distance from one sensor node to the rest, there is no coupling among them. However, the sensor nodes relations through the circuit of the network branch introduce a dependency problem, which is similar to the cross-coupling effect of the SIMO–WPT systems studied in the literature.

Currently, SIMO–WPT systems are a hot topic in the WPT research area, as was appointed previously in Section 1. The most effective method to maximize the energy transmission in a SIMO–WPT system is to remove elements from the charging system. For example, if there is a set of electric cars parked together in charging state using a SIMO–WPT system, when one of those cars leaves the parking, the remaining cars receive more energy [21,22].

Certainly this solution would involve removing sensor nodes from the network branch. However, each sensor node included in the sensor network is specified by the operating conditions of the measurement application. Because of this, in the sensor network there are no expendable elements. Therefore, this solution cannot be applied.

Other authors increase the efficiency of the charging system minimizing the cross-coupling effect using a time-sharing approach [20]. These authors control each charging element disconnecting certain sensor nodes while charging others. The disconnection is made using a bridge of rectifiers. A centralized control circuit manages the whole process. This last requirement implies a direct connection, that is a control communication channel (CCC), between each sensor node and the centralized control. They use a wired solution to implement CCC. So, in order to use this approach in our application, it is mandatory to increase substantially the circuital complexity of the solution adding an extra wireless connection to implement CCC. Obviously, increasing the circuits complexity also makes the power consumption higher.

In our research, we exploit the disconnection idea using a novel simpler solution than a bridge of rectifiers and propose a distributed control approach explained in the following.

#### 3.2.1. Sensor Node Disconnection

All the components’ values of the network branch are optimized to obtain a maximum energy transfer at the desired frequency. Each antenna is compensated with a capacitor. For example, if we modify the value of the compensation capacitor of a sensor node reception antenna from its optimal value, the sensor node efficiency is reduced. In other words, if we change the central frequency of the band-pass filter which defines the sensor antenna and its compensator capacitor, we are reducing the load effect of this sensor node over the network branch.

**Figure 6:**
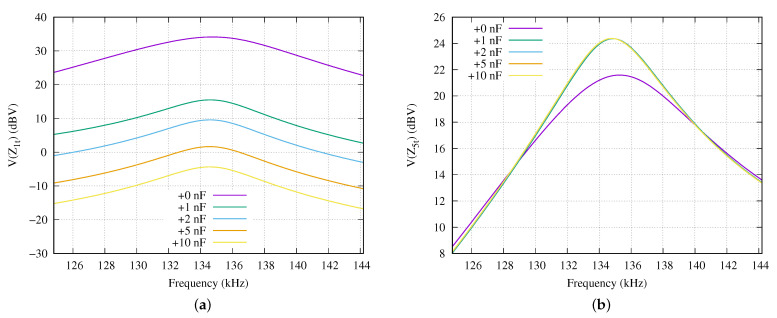
Frequency response of the sensor node load when the compensator capacitor is increased 1, 2, 5 and 10 nF in comparison with its optimal value: (**a**) First sensor node (i=1) changing its compensation capacitor and (**b**) deepest sensor node (i=5).

Now, we are going to evaluate the frequency response of the circuit presented in Figure 2 using the values from Table 1 for the deepest sensor node i=5 and for the closest to ocean surface i=1 sensor nodes when this last one modifies its reception compensation capacitor $C_{1t}$. Figure 6a presents the behavior in frequency of the voltage applied to the load impedance of the most shallow sensor node, when its compensation capacitor differs from its optimal value.

The $C_{1t}$ capacitor optimal value (see +0 nF in Figure 6, for more details) is compared with increasing values of 1, 2, 5 and 10 nF. The maximum response is located in 134.2 kHz. If the optimal value is set in $C_{1t}$, the obtained voltage is 34.06 dBV. When this capacitor is increased in 1 nF, the obtained voltage in most shallow sensor node is 15.41 dBV. This means a reduction of the received voltage of 18.65 dB. In case of applying an increment in $C_{1t}$ of 10 nF, the attenuation is 38.49 dB in the received voltage at $Z_{1t}$. A consequence of those modifications of the $C_{1t}$ capacitor value is that the voltage received in deepest sensor node $V(Z_{5t})$ is altered from 21.3 dBV when the optimal value of $C_{1t}$ is set to 24.11 dBV in case of an increment of 1 nF. This means 2.81 dB more voltage applied to this deepest sensor node. However, further increments of $C_{1t}$ do not increase this gain. For example, increasing $C_{1t}$ in 10 nF, improves the voltage in 0.06 dB.

#### 3.2.2. Disconnection Strategy

As summary, all solutions presented in the literature are based on a centralized control of the WPT system resources. However, the implementation of those strategies produces non-optimal solutions in this type of applications. For example, using a wired solution in this application makes it lose its key short-circuit tolerance feature [14], and the wireless solution requires extra circuit infrastructure increasing the power consumption.

A decentralized solution implies the existence of a minimal distributed intelligence with which to take decisions in each sensor node. In our application we have a microcontroller in the SoC. On the other hand, the nature of the sensor nodes network causes its behavior to be deterministic. That is, given a measurement, it is possible to determine all the time parameters involved because there are no random events such as alarms.

Taking into consideration the status of the SoC in each sensor node in terms of power consumption, we can conclude that most of the time the sensor nodes are charged and waiting for the deepest one. The solution proposed in this paper is to self-disconnect/detune each sensor node during this charging and waiting time $t_{b}$.

#### 3.2.3. Sensor Node Circuit Modification

**Figure 7:**
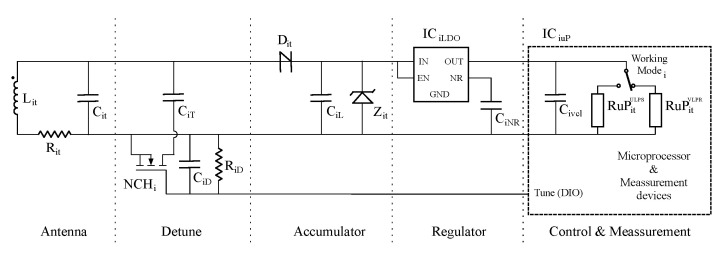
Proposed batteryless sensor node equivalent circuit.

Figure 7 presents the equivalent schematics of the used sensor node to test our proposed charging method. This circuit includes a new stage called Detune in comparison with the original circuit introduced in Figure 4. The new stage is inserted in parallel between the Antenna and Accumulator stages. It also requires a control line from the microprocessor.

The proposed stage contains the detuning capacitor $C_{iT}$ that corresponds to the capacitance used to increase the coupling capacitor $C_{it}$. $C_{iT}$ is connected serially to a n–channel MOSFET $NCH_{i}$. In order for this transistor to operate correctly, it is mandatory to include the gate decoupling capacitor $C_{iD}$ and the pull–down resistor $R_{iD}$. Finally, a digital output (DIO) port of the microprocessor controls the gate of the transistor.

It is remarkable that, after programming the value of the labeled Tune DIO of the microprocessor, this value is held in ULPS mode. In addition, in order to manage correctly the tunning and detuning process, it is mandatory that the microprocessor initializes its digital outputs to zero after a power-on reset (POR). Finally, Table 4 provides the values of all components used in this new stage in addition to the presented in Table 2.

At this point, with the hardware modifications of the sensor node and its behavior during the charging process presented, we are able to evaluate its usefulness in several experimental scenarios.

## 4. Experiments

### 4.1. Single Shot Measurement

This experiment consists in executing a measurement in a single specific deep sensor node of the network branch. As mentioned in [17], the received energy in the sensor nodes is less as depth increases. Therefore, the worst corner case is defined by the single shot measurement of the deepest sensor node. In order to check the behavior of our proposed charging method, we use a network branch with five sensor nodes (N=5).

**Figure 8:**
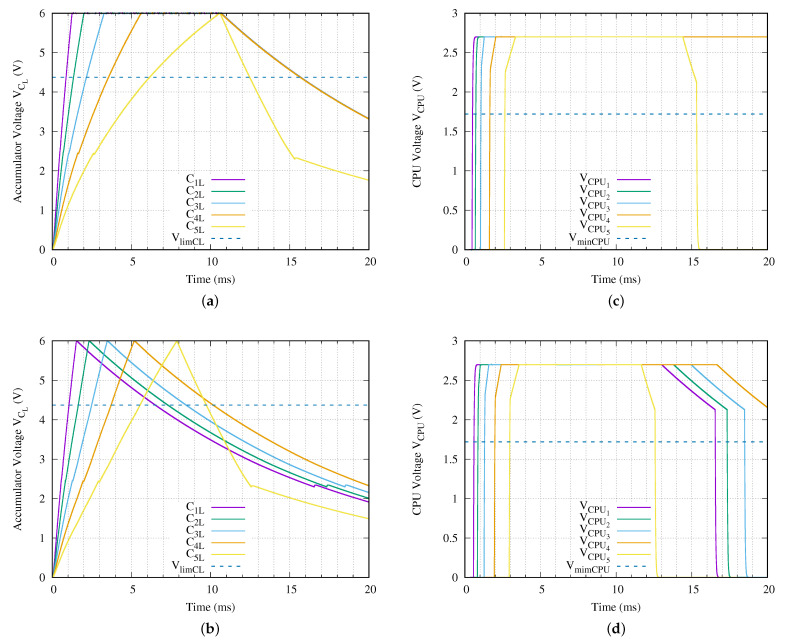
Energy accumulator capacitor and CPU voltages of each sensor node for a network branch with five levels (*N* = 5) using (**a**,**c**) Traditional Charging Method and (**b**,**d**) Proposed Charging Method.

Figure 8 presents the voltage of the energy accumulator and its CPU of all sensor nodes in the proposed experiment. The charging behavior using the traditional method is shown in Figure 8a. In this experiment, we apply a SCT of $V_{in}$ equal to 5 V peak-to-peak (V${}_{pp}$). This charging tone is applied until the fully charged state of the deepest sensor node is obtained. The required time is 10.581 ms.

We assume that all sensor nodes energy accumulators are uncharged, that is $V_{CL}$ = 0 V. Then, the SCT is applied. All sensor nodes begin to charge their energy accumulator capacitors as shown in Figure 8a from time 0 to 10.581 ms. In each sensor node, once the voltage regulator activates its output, the microprocessor starts to run (see Figure 8c for more details). However, once each sensor node microprocessor executes its power on reset, it goes to ULPS mode. All sensor nodes continue in this state until the end of the experiment except the deepest sensor node. When the deepest sensor node reaches the fully charged state, the measurement process begins. The execution of the measurement only uses the stored energy.

From our experiments we determined that the sensor node microprocessor and the associated measurement devices consume a current of 120 nA when the power supply voltage is 2.7 V. This is equal to a resistance of 22.5 MΩ. On the other hand, this current is equivalent to 180 μA in average when all measurement devices and the microprocessor are running along a measurement process. The difference between current consumptions is the reason for having two different discharging rates of $t_{d}$ after the 10.58 ms (see Figure 8a). This behavior fulfills the ISO11784/11785 standard presented in Section 2.2.

Figure 8 presents the behavior of the single shot measurement using our proposed charging method. Assuming the same conditions as the traditional method, the sensor nodes have 0 V in their energy accumulators. A SCT tone, with an amplitude of 5 V${}_{pp}$, begins to charge each sensor node accumulator capacitor $C_{L}$. In this case, the duration of this charging tone is 7.877 ms which corresponds with the required time to reach the fully charged state of the deepest sensor node. In a similar way to traditional method, as soon as the voltage regulator of each sensor node activates its output, the microprocessor goes to ultra low power stop mode. However, following our proposal, in addition to going to ultra low power stop, it detunes their reception antenna. Moreover, the deepest sensor node starts to execute the experiment once its energy accumulator capacitor $C_{L_{5}}$ reaches the fully charged state.

When the proposed method is used, the execution of the measurement is started 7.877 ms after applying the SCT. It represents a 25.56 % of less required time in comparison with the traditional charging method. Table 5 summarizes the charging times for all sensor nodes of the network branch under test. In this table, in addition to the sensor node id, the charging times using the traditional and the proposed method, the last two columns show the difference between both methodologies in terms of reduced time and its percentage.

Including extra components to the reception circuit of the sensor node has a cost in terms of effective load. Looking to the last two columns in Table 5, we observe the load effect of the added auxiliary circuitry over the charging process. The three less deepest sensor nodes require from 0.231 ms to 0.335 ms, due to this reason. In this experiment setup, using the proposed method and the enumerated commercial off-the-shelf (COTS) devices, the advantage of the proposed method is greater than the loss, due to the extra circuit in the two deepest sensor nodes.

From the data comparisons, it is deduced that if the required time to execute the Single Shot Experiment in the shallow nodes is taken into account, to use the proposed method makes it nonoptimal. However, if we take into consideration the reduction of the maximum required time and the dimension of the non-optimal against the optimized sensor nodes, using our charging method increases the required time only 0.336 ms in the worst case and decreases this time in 2.705 ms for the best case.

### 4.2. Snapshot Measurement

One of the most common experiments in oceanic underwater cages is to obtain a snapshot of the complete underwater sensor network. The objective of this experiment is to know the status of the deployed infrastructure or their environmental variables. Basically, it involves measuring acceleration, turbidity or temperature among others, simultaneously, in all sensor nodes. From the point of view of the batteryless sensor nodes, this experiment is executed in two basic stages: charge and measure.

The worst case scenario is defined when the measurement process requires a large part or all of the energy stored in their accumulators. In this corner case, all sensor nodes in the network must be fully charged to execute the measurement. It is well known that a greater number of sensor nodes reduces the charging capability of the network, i.e., fully charging time or maximum available charge.

In order to check the underwater sensor network, we propose to evaluate a network branch with ten batteryless sensor nodes (*N* = 10) when a SCT ($V_{in}$) of 24, 18, 10 or 5 V${}_{pp}$ is applied.

#### 4.2.1. Changing the Sensor Node

**Figure 9:**
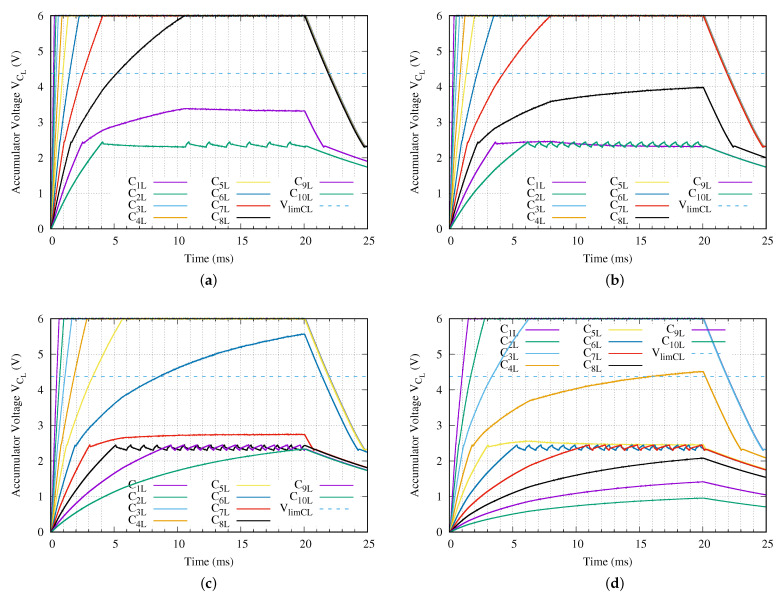
Energy accumulator capacitor voltage of each sensor node for a network branch with ten levels (*N* = 10) when a (**a**) $V_{in}$ = 24 V, (**b**) $V_{in}$ = 18 V, (**c**) $V_{in}$ = 10 V or (**d**) $V_{in}$ = 5 V peak-to-peak sinusoidal charging tone is applied.

The main key here is to determine the number of working nodes which reaches the maximum charging voltage in the setup proposed. Figure 9 presents the voltage of the energy accumulator capacitor $C_{L}$ for all sensor nodes in the experiment proposed. As was appointed previously, the maximum charging voltage is 6 V. Figure 9a provides the behavior of the traditional method when it is applied a charging tone of $V_{in}$ = 24 V${}_{pp}$. In this case, the circuit charges up to seven sensor nodes in less than 5 ms ($t_{a_{i}}\;\leq \;$4.12 ms, ∀i∈[1,7]). In addition, the eighth sensor node requires $t_{a_{8}}$=10.53 ms to be fully charged. Finally, the last two sensor nodes never reach the fully charged state.

In Figure 9, we show the effect of the activation of the LDO output over the charging process (see label 4 in Figure 3b). Obviously, the less energy available, the greater the effect. This issue is more evident in sensor nodes 8 and 9, in Figure 9a. Once the LDO activates its output, the slope of the energy accumulator capacitor voltage decreases substantially for those mentioned sensor nodes. Certainly, this behavior is attenuated depending on the energy available at the level of the network branch, as is the case of the first seven sensor nodes. On the other hand, the worst case is the energy accumulator capacitor voltage for the last sensor node in Figure 9a, which exhibits a ripple behavior for this reason.

Figure 9b–d present the behavior of all sensor nodes of the network branch when the SCT $V_{in}$ is set to 18, 10 and 5 V${}_{pp}$, respectively. As expected, if the input voltage is reduced, less sensor nodes reach the fully charged state. For example, when the input voltage is set to 18 V${}_{pp}$ the last three sensor nodes do not get fully charged. In case $V_{in}$ equals 5 V${}_{pp}$ only the first three sensor nodes reach the fully charged state within the 20 ms of the input charging tone. The fourth sensor node does not reach the fully charging voltage despite extending the input charging tone up to 50 ms.

Table 6 presents the required times to reach the fully charged state $t_{a}$, when the proposed input voltages are applied during 20 ms. A higher applied voltage implies faster fully charging time. This is the reason for increasing from 0.2943 ms when 24 V${}_{pp}$ is applied against the required 1.491 ms when only 5 V${}_{pp}$ is available. The time lap between both first sensor nodes is 1.1967 ms. It is close to 5 times longer, but the applied power is reduced up to a 79.17 %. It is also remarkable that the last sensor node which reaches the fully charged requires at least twice as long as the immediately preceding sensor node. For example, in the last column (5 V${}_{pp}$) of this Table 6, the deepest sensor node that reaches the fully charged state requires 6.29 ms and the previous one takes only 2.7749 ms.

In summary, using the traditional method, we provide energy to 7 sensor nodes within the first 5 ms when a tone of 24 V${}_{pp}$ is used, and the total available sensor nodes are 6, 4 and 2 in case of applying 18, 10 and 5 V${}_{pp}$, respectively, for those 5 ms. Taking $V_{in}$ = 24 V${}_{pp}$ as reference, the voltage reduction decreases the power of the applied tone a 43.75 %, 82.63 % and a 95.66 % for later voltages, respectively.

#### 4.2.2. Measurement Execution

**Figure 10:**
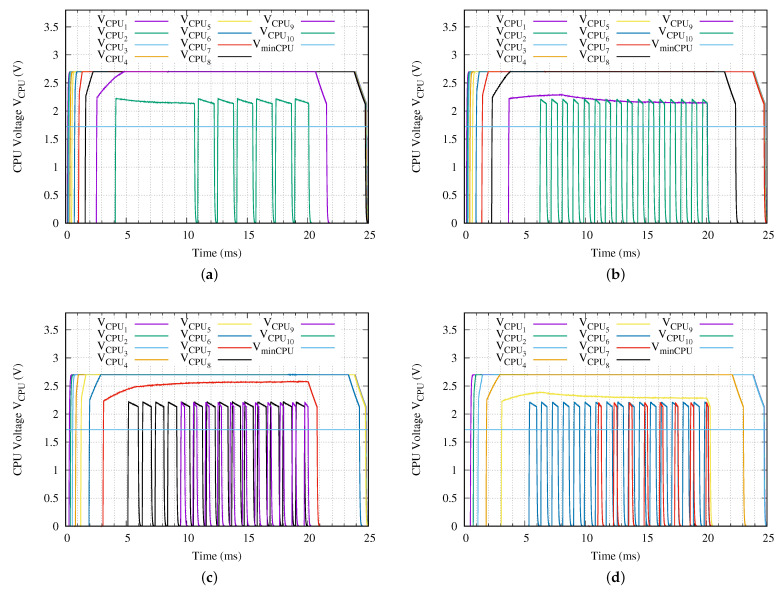
Voltage applied to each CPU for a network branch with ten sensor nodes (*N* = 10) when a (**a**) $V_{in}$ = 24 V, (**b**) $V_{in}$ = 18 V, (**c**) $V_{in}$ = 10 V or (**d**) $V_{in}$ = 5 V peak-to-peak sinusoidal charging tone is applied.

Once the fully charging time $t_{a}$ is determined, it is mandatory to study the supply voltage applied to the CPU of the sensor node in order to calculate the available measurement time. Certainly, the more time available, the better the solution. However, because we are planning to perform a single measurement, there is a minimum required time to set up the included instrument devices and run a measurement ($min(t_{meas})$). In this experiment, since the sensor node microprocessor is running at 8 MHz, we have determined that it is 1.0 ms. However, in order to evaluate the sensor network, we set the minimum required measurement time $min(t_{meas})$ to 2.0 ms. Figure 10 presents the voltage applied to each CPU of the proposed experiment.

From the point of view of the CPU, we observe in Figure 10 the erratic behavior of the LDO output voltage for the deepest sensor nodes. For example, when it is applied a $V_{in}\;=$ 24 V${}_{pp}$ as shown in Figure 10a, the CPU is turned on and off several times during the presence of the SCT. Other undesirable effect is depicted on Figure 10b, where the voltage applied to the eighth sensor node, $V_{CPU_{8}}$ turns on and off the CPU correctly, but it never reaches the 2.7 V stable voltage. Table 7 summarizes the most important time parameters from Figure 10.

From the point of view of the voltage applied to the microprocessor in comparison with the fully charging time $t_{a}$, Table 7 indicates that it is possible to keep the microprocessor voltage stable despite the accumulator capacitor is not fully charged. As was appointed in the previous section, the more time available, the better the solution. The stable condition in the microprocessor voltage supply is mandatory for obtaining a right measurement. This table shows that, for example, when 24 V${}_{pp}$ is applied in $V_{in}$, only the deepest sensor node does not work as expected. However, if we remember the fully charging time $t_{a}$ for the second deepest sensor node from Table 6, we can see that in this experiment the sensor node accumulator never reaches the fully charged state. In these conditions, it is not possible to use this second deepest sensor node to execute a measurement.

Table 7 identifies three different failures that appear. The first identified failure N27 is that the supply voltage of the microprocessor does not reach 2.7 V stably within a SCT for $t_{SCT}$ of $t_{a}$ + $t_{b}$ = 20 ms. In general this malfunction arises when the charging capabilities of the network branch are slightly lower than the equivalent load of a sensor node. Once this behavior occurs, all the deeper sensor nodes do not work correctly. Despite the failure, in the best case, the processor turns on and after a period of time it turns off. However, in the worst case, the processor turns on and off repeatedly (TOR). Finally, Table 7 identifies those sensor nodes which do not receive enough power to turn on their microprocessor with a dash sign.

Once it is determined the fully charging time $t_{a}$ for all sensor nodes and what sensor nodes can be used to execute a measurement, we can reduce the duration of the SCT ($t_{a}$ + $t_{b}$). Because we assume that the measurement process only begins when the energy storage capacitor of the last sensor node reaches the state of fully charged, we define the remaining time $t_{b_{n}}$ as zero in the deepest working sensor node *n* in a network branch with *N* sensor nodes. The last step in this optimization is to check the stability of the voltage applied to the sensor node microprocessor along the measurement execution within the discharging time $t_{d}$. We define this time as:

(2) $$t_{d_{stable}}=t_{on}+t_{tr}+t_{stable}-t_{a}-t_{b}$$

Table 8 summarizes the main features of the optimized network branch with ten sensor nodes when several voltages of the SCT are applied. The first column presents the applied voltage in volts peak-to-peak. The second column identifies the deepest sensor node what works correctly and therefore a measurement can be executed. In the next column, the charging time of the deepest working sensor node is given. Finally, the last column provides the maximum execution time $t_{stable}$ in which the sensor node can execute a measurement.

The maximum execution time for the measurement process can be slightly greater than 3.8 ms in all cases. It is basically because we set the requirement that the experiments begin their execution when the energy accumulator is fully charged. Note that we set the target time for a desired measurement in 1 ms plus other millisecond extra for safety purposes. Therefore, now we have two possibilities to optimize even more the process. One is to evaluate the minimum charging level where the energy available provides the stable voltage along the measurement process. The other one is to determine the capacitance value of the energy accumulator capacitor which allows the measurement process. This last solution implies a hardware modification when a new measurement execution time is required. In this research, we focus our optimization effort in the first option. This means optimizing the charging time via reducing the required charging level for a given energy accumulator capacitor.

Taking into consideration that we are discharging a capacitor, the minimum voltage ${\bar{V}}_{CL}$ which allows to execute a measurement is modeled as:

(3) $${\bar{V}}_{C_{L}}=V_{limCL}\;e^{-\frac{t_{m}}{R_{L}\times C_{L}}}$$

where $t_{m}$ is the duration of the measurement execution (2 ms), $R_{L}$ is the equivalent supported load (15 kΩ), $C_{L}$ is the value of the energy accumulator capacitor (33 nF) and ${\bar{V}}_{CL}$ is the voltage level just after finishing the execution of the measurement. This ${\bar{V}}_{CL}$ is obtained from the LDO datasheet or based on simulation. It is defined as the voltage at the energy accumulator capacitor just before the stable condition in the regulated voltage applied the the microprocessor is lost. ${\bar{V}}_{CL}$ is identified in Figure 3b as label $9^{\prime }$. In our experiments, we used a ${\bar{V}}_{CL}$ equal to 2.876 V. Finally, the calculated $V_{limCL}$ is 4.374 V.

### 4.3. Optimized Snapshot Measurement

#### Charging the Sensor Node

**Figure 11:**
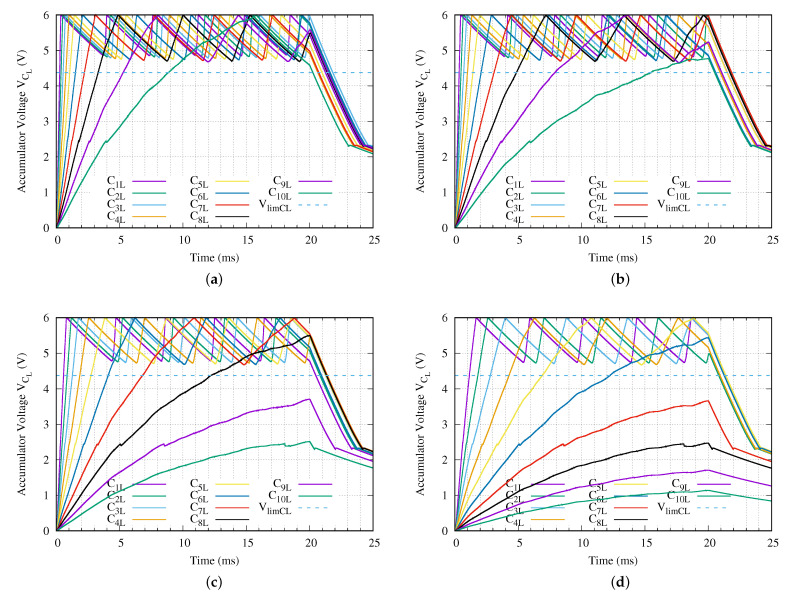
Energy accumulator capacitor voltage of each sensor node for a network branch with ten levels (*N* = 10) when a (**a**) $V_{in}$ = 24 V, (**b**) $V_{in}$ = 18 V, (**c**) $V_{in}$ = 10 V or (**d**) $V_{in}$ = 5 V peak–to–peak sinusoidal charging tone is applied using the proposed method.

In this point, the charge of the energy storage capacitor when applying the proposed method is studied in detail. We take into consideration that from the point of view of the measurement, it can be executed if the voltage capacitor $V_{limCL}$ reaches at least 4.374 V. Figure 11 presents the behavior of the energy accumulator capacitor $C_{L}$ for all sensor nodes in the previous proposed experiment when our method is applied. Figure 11a–d present the $C_{L}$ voltage in each sensor node when the sinusoidal charging tone $V_{in}$ is 24, 18, 10 and 5 V${}_{pp}$, respectively.

At first, we observe in Figure 11a that all signals reach the fully charged voltage within the 20 ms duration of the sinusoidal charging tone when the voltage applied is 24 Vpp. In case of a $V_{in}$ equal to 18 V${}_{pp}$, only the deepest sensor node does not reach the fully charging voltage (6 V). However, it achieves a voltage greater than the minimum required voltage to execute correctly the measurement $V_{limCL}$ as shown in Figure 11b. When the voltage applied with the SCT is 10 or 5 V${}_{pp}$, the third and fifth deepest sensor nodes do not hit the desired fully charged voltage respectively (see Figure 11c,d for more details). However, the third deepest sensor node when $V_{in}$ is 10 V${}_{pp}$ and the fifth deepest sensor when $V_{in}$ is 5 V${}_{pp}$ beat the minimum required voltage to execute the measurement.

Table 9 presents the required times to reach the fully charged state $t_{a}$ when we apply our proposed method during 20 ms of the SCT with several voltages. In case of using a $V_{in}$ equal to 24 V${}_{pp}$ when our proposed method is applied, all ten sensor nodes of the network branch reach the fully charging condition within the 20 ms duration of the charging tone defined in the ISO 11784/11785 HDX. As shown in the second column of this Table 9, the slowest, that is the deepest, sensor node hits the fully charging state in less than 15.8 ms. In case of using a $V_{in}$ equal to 18 V${}_{pp}$, the deepest sensor node of the network branch does not reach the fully charging state along the 20 ms charging tone. However, this sensor node gets the minimum charging voltage to execute the measurement in 15.5 ms. So even though not all sensor nodes reach the fully charged condition, it is possible to execute the measurement correctly in the complete network branch.

When the SCT amplitude is 10 V${}_{pp}$, the three deepest sensor nodes never reach the fully charged condition and only the third deepest sensor node gets a voltage greater than $V_{limCL}$. In this case, the measurement can be executed in the eight sensor nodes. Finally, when only 5 V${}_{pp}$ are applied, the five less deepest sensor nodes reach the fully charging condition. In addition, the fifth deepest sensor node beats the $V_{limCL}$. It takes only 12.3 ms of the SCT.

**Figure 12:**
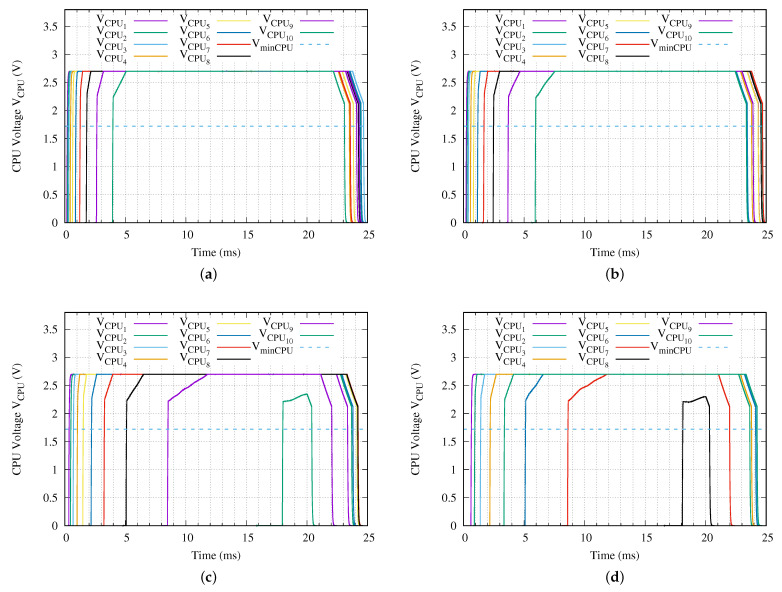
Voltage applied to each CPU for a network branch with ten sensor nodes (*N* = 10) when a (**a**) $V_{in}$ = 24 V, (**b**) $V_{in}$ = 18 V, (**c**) $V_{in}$ = 10 V or (**d**) $V_{in}$ = 5 V peak–to–peak sinusoidal charging tone is applied using the proposed method.

Figure 12 presents the voltage applied to each microprocessor of a network branch with ten sensor nodes when it is used our proposed charging method. We observe in Figure 12, that all the processors have a stable voltage within the first 5 ms of the SCT with $V_{in}$ equal to 24 V${}_{pp}$. This stable voltage is reached in less than 8 ms when the applied voltage is reduced to 18 V${}_{pp}$. The deepest sensor node turns on and then turns off close to the 18 ms and the 20.5 ms time instants, respectively, and never reaches the stable condition when 10 V${}_{pp}$ is set in $V_{in}$. Finally, when it is applied for 5 ms the deepest sensor node never turns on. In addition, the second deepest sensor node turns on and off in a short time period of 2 ms. However, this sensor node never reaches the stable voltage condition.

Table 10 presents the most important time parameters from Figure 12. As was appointed in the evaluation of the charging time of the energy accumulator capacitor in previous paragraphs, when the input SCT has an amplitude of 24 V${}_{pp}$, all the ten sensor nodes of the network branch under test run correctly. The deepest sensor node is the slowest and it requires at least 5.059 ms to reach the stable voltage. In a similar way, when $V_{in}$ is set to 18 V${}_{pp}$ the deepest sensor node requires only 7.538 ms to reach the stability in its microprocessor voltage. It is remarkable that, for all the tests performed using our proposed charging method, the voltage applied to the microprocessor does not turn on and off repetitively like it is observed when using the traditional charging method.

Combining the fully charging times information from Figure 11, the microprocessors stable running times from Table 10 and using Equation (2), it is possible to determine the minimum charging time that requires the network branch to execute correctly a snapshot measurement. Table 11 presents these data.

In comparison with the traditional approach summarized in Table 8, our proposed charging method always enables more sensor nodes to execute the measurement. For example, in case of using a $V_{in}$ of 24 V${}_{pp}$, all the sensor nodes can execute the experiment when our proposal is used in contrast to the traditional method that is able to charge correctly 2 sensor nodes only. Certainly, increasing of the number of working correctly sensor nodes requires extra time in comparison with the traditional method. However, with the traditional method it is impossible to run the measurement on those additional sensor nodes, despite increasing the duration of the SCT up to the ISO 11784/11785 limit of 50 ms.

The great advantage is produced when $V_{in}$ is set to only 5 V${}_{pp}$. In this case, the proposed method allows one to execute the measurement in 5 sensor nodes with a fully charged energy accumulator capacitor in comparison with the traditional method that allows three sensor nodes to run at most in the same conditions. In addition, as shown in the last two columns of this Table 11, it is possible to execute the measurement correctly in an extra deeper node than the ones shown in column two if we assume that the charged voltage of the energy accumulator beats the $V_{limCL}$ without reaching the fully charged state. Taking into consideration this condition, the total number of available sensor nodes are 10, 8 and 6 when 18, 10 and 5 V${}_{pp}$ are applied respectively in $V_{in}$. Taking into account a maximum distance of 6 m between the sensor nodes and the hub, ten sensor nodes imply a network branch 60 m deep.

## 5. Conclusions

This work aims to increase the total number of batteryless sensors deployed in a practical underwater sensor network. On the other hand, the second objective of this proposal is to reduce the power consumption of the sensor network in comparison with the approach found in the literature. We presented a distributed charging strategy based on the sensor nodes self disconnection principle. The implementation of this new charging strategy only requires a slight modification of the sensor nodes antenna circuitry and the solution adopted still meets the ISO 11784/11785 HDX standard. Therefore, the network infrastructure remains unaltered.

The sensor node power consumption optimization is performed and the design considerations discussed in detail. The sensor node disconnection feature, the new strategy and its implementation using commercial devices are presented. Two real corner cases are used to evaluate the proposed batteryless sensor node network charging method.

One corner case consists in charging the deepest sensor node and the other one is to charge all sensor nodes at the same time. From the experiment of charging the deepest sensor node, the obtained results indicate that the proposed solution is a 25% faster in comparison with the approach found in the literature. This percentage represents around 2.7 ms less in required time. For the less deepest sensor node our approach requires close to 21% longer. However, this only represents 0.26 ms more. The advantage is greater as the sensor node is located deeper.

On the other hand, when all sensor nodes must be charged, the traditional method is limited to charging five sensor nodes when 10 V peak to peak are used as charging tone of the sensor network. In this case the fully charged condition is reached after 5.68 ms approximately. However, when the proposed charging method is used, this time is reduced to 3.83 ms, which is 32.5% less time than the traditional method. In addition, it is possible to charge two extra deepest nodes if we assume a greater charging time. These two extra sensor nodes cannot be charged using literature approach.

Finally, the total number of fully charged sensor nodes is studied in terms of the applied sinusoidal charging tone voltage. Obviously, to apply a higher voltage increases the number of fully charged sensor nodes. Additionally, the sensor nodes close to the ocean surface decrease their fully charging time. However, it is possible to use 5 V peak-to-peak when our solution is applied to charge up to seven sensor nodes. In the same conditions, with the traditional method, it is only possible to charge up to three sensor nodes. This is half of that obtained in our approach.

## Figures and Tables

**Table 1:** Experiment Setup for network branch with five stages used as example.

Parameter	Value
*N*	5
$L_{ia}$ and $L_{ib}$	452 μH
$M_{iab}$	316.16 μH
$M_{iat}$ and $M_{jbt}$	73.5 μH
$C_{ja}$ and $C_{ib}$	3.1117 nF
$C_{5a}$	3.0978 nF
$L_{it}$	2.66 mH
$C_{it}$	526.489 pF
$Z_{it}$	15k Ω
$R_{ia}$ and $R_{ib}$	620.16 mΩ *^1^

i∈[1,5] and j∈[1,4]. *^1^ using 23 AWG standard.

**Table 2:** Sensor node components values.

Parameter	Value	Stage	Description
$L_{it}$	2.725 mH	Antenna	Antenna
$R_{it}$	26.9 Ω		Antenna Parasitic
$C_{it}$	516.141 pF		Tunning Capacitor
$D_{it}$	$V_{f}$ = 715 mV	Accumulator	Rectifier
$C_{iL}$	330 nF		Energy Accumulator
$Dz_{it}$	$V_{z}$ = 6 V		Energy Limiter
$IC_{iLDO}$	TPS71727	Regulator	LDO Regulator
$C_{iNR}$	10 nF		Noise Reduction
$C_{iRuP}$	10 nF	Control & Meassurement	μP Equivalent
$RuP_{it}^{ULPS}$	22.5 MΩ		ULPS Modeling
$RuP_{it}^{VLPR}$	15 kΩ		VLPR Modeling
$IC_{iuP}$	MKL17Z256		Microprocessor

i∈[1,N] and *N* is the number of sensor nodes.

**Table 3:** Basic parameters from components required to determine the ISO 11784/5 standard usage.

Parameter	Value	Description
$V_{runCPU}$	2.7 V	Maximum VLPR power supply voltage
$V_{MaxC_{L}}$	6 V	Maximum LDO regulated input
$V_{minLDO}$	2.45 V	Minimum LDO regulated input
$V_{minCPU}$	2.25 V	Minimum LDO regulated output
$max(t_{SCT})$	50.0 ms	Maximum SCT time
$typ(t_{SCT})$	20.0 ms	Typical SCT time
$min(t_{SCT})$	5.0 ms	Minimum SCT time

**Table 4:** Proposed sensor node components values for the Detune stage.

Parameter	Value	Description
$C_{iT}$	10 nF	Detunning
$NCH_{i}$	BS170p	Switch
$C_{iD}$	1 nF	Decoupling Switch
$R_{iD}$	2.47 MΩ	Pull–down gate

i∈[1,N] and *N* is the number of sensor nodes.

**Table 5:** Charging time $t_{a}$ obtained using the traditional charging technique and $t_{a}^{opt}$ charging time when the proposed charging method is applied.

ID	$t_{a}$	$t_{a}^{\mathrm{opt}}$	diff	diff (%)
1	1.267	1.528	+0.261	+20.59
2	2.005	2.340	+0.335	+16.70
3	3.279	3.510	+0.231	+7.04
4	5.682	5.195	–0.487	–8.57
5	10.582	7.877	–2.705	–25.56

**Table 6:** Fully charging time $t_{a}$ in a network branch with ten sensor nodes in terms of the voltage amplitude $V_{in}$ of the applied sinusoidal charging tone.

ID	$t_{a}$				
	$V_{\mathrm{in}}$	24	18	10	5
1		0.294	0.368	0.666	1.491
2		0.413	0.538	1.036	2.774
3		0.599	0.801	1.672	6.290
4		0.890	1.225	2.858	NF *^1^
5		1.387	1.978	5.679	NF
6		2.297	3.507	NF	NF
7		4.121	7.978	NF	NF
8		10.531	NF	NF	NF
9		NF	NF	NF	NF
10		NF	NF	NF	NF

NF: No Fully charged in *t_a_* + *t_b_* = 20 ms. *^1^: No Fully charged in *t_a_* + *t_b_* = 20 ms.

**Table 7:** Fully charging time $t_{a}$ in a network branch with ten sensor nodes in terms of the voltage amplitude $V_{in}$ of the applied sinusoidal charging tone.

$V_{\mathrm{in}}$	ID	$t_{\mathrm{on}}$	$t_{\mathrm{on}}$ + $t_{\mathrm{tr}}$	$t_{\mathrm{run}}$	$t_{stable}$
24 V${}_{pp}$	1	0.177	0.385	24.675	23.560
	2	0.222	0.457	24.619	23.478
	3	0.289	0.520	24.538	23.400
	4	0.385	0.615	24.427	23.291
	5	0.534	0.770	24.265	23.122
	6	0.752	1.010	24.025	22.861
	7	1.100	1.376	23.657	22.476
	8	1.645	2.273	23.100	21.566
	9	2.568	4.844	18.989	15.801
	10	TOR	N27	TOR	N27
18 V${}_{pp}$	1	0.231	0.433	24.606	23.498
	2	0.289	0.515	24.536	23.404
	3	0.372	0.607	24.439	23.298
	4	0.559	0.739	24.237	23.152
	5	0.714	0.929	24.070	22.948
	6	1.020	1.249	23.740	22.605
	7	1.504	1.982	23.241	21.854
	8	2.316	3.799	20.083	17.697
	9	3.703	N27	16.365	N27
	10	TOR	N27	TOR	N27
10 V${}_{pp}$	1	0.335	0.546	24.475	23.358
	2	0.440	0.683	24.358	23.208
	3	0.604	0.854	24.180	23.022
	4	0.872	1.121	23.896	22.742
	5	1.305	1.687	23.451	22.160
	6	1.990	2.912	22.275	20.449
	7	3.145	N27	17.617	N27
	8	TOR	N27	TOR	N27
	9	TOR	N27	TOR	N27
	10	—	—	—	—
5 V${}_{pp}$	1	0.559	0.790	24.210	23.072
	2	0.791	1.042	23.965	22.806
	3	1.178	1.525	23.563	22.311
	4	1.855	2.955	22.190	20.184
	5	3.118	N27	17.183	N27
	6	TOR	N27	TOR	N27
	7	TOR	N27	TOR	N27
	8	—	—	—	—
	9	—	—	—	—
	10	—	—	—	—

N27: 2.7V no reached in *t_a_* + *t_b_* = 20 ms. TOR: Turn on and off repeatedly. —: Never turn on.

**Table 8:** Network branch optimized charging times for the given input voltages and maximum measurement times.

$V_{\mathrm{in}}$	*n*	$t_{a_{n}}$	$t_{d_{stable}}$
24	8	10.531	3.839
18	6	7.978	3.854
10	5	5.679	3.847
5	3	6.290	3.836

**Table 9:** Fully charging time $t_{a}$ in a network branch with ten sensor nodes in terms of the voltage amplitude $V_{in}$ of the applied sinusoidal charging tone using the proposed disconnection method.

ID	$t_{a}$				
	$V_{\mathrm{in}}$	24	18	10	5
1		0.368	0.455	0.828	1.684
2		0.526	0.677	1.187	2.622
3		0.754	0.960	1.747	3.980
4		1.020	1.353	2.570	6.274
5		1.438	1.959	3.831	10.777
6		2.097	2.898	6.208	NF *^1^
7		3.110	4.480	10.905	NF
8		4.878	7.190	NF *^2^	NF
9		8.041	13.696	NF	NF
10		15.799	NF *^3^	NF	NF

NF: No Fully charged in *t_SCT_* = 20 ms. *^1^: ${\bar{V}}_{C_{L}}$ in *t_SCT_* = 12.3 ms. *^2^: ${\bar{V}}_{C_{L}}$ in *t_SCT_* = 12.2 ms. *^3^: ${\bar{V}}_{C_{L}}$ in *t_SCT_* = 15.5 ms.

**Table 10:** Fully charging time $t_{a}$ in a network branch with ten sensor nodes in terms of the voltage amplitude $V_{in}$ of the applied sinusoidal charging tone using the proposed charging method.

$V_{\mathrm{in}}$	ID	$t_{\mathrm{on}}$	$t_{\mathrm{on}}$ + $t_{\mathrm{tr}}$	$t_{\mathrm{run}}$	$t_{stable}$
24 V${}_{pp}$	1	0.206	0.421	22.890	21.762
	2	0.260	0.503	23.237	22.080
	3	0.348	0.590	23.224	22.069
	4	0.476	0.723	23.353	22.192
	5	0.662	0.900	23.440	22.286
	6	0.981	1.160	23.264	22.168
	7	1.264	1.524	23.049	21.875
	8	1.809	2.153	22.598	21.338
	9	2.644	3.198	21.820	20.353
	10	3.970	5.079	20.708	18.684
18 V${}_{pp}$	1	0.243	0.474	23.114	21.968
	2	0.313	0.563	23.112	21.948
	3	0.425	0.677	23.021	21.855
	4	0.601	0.847	23.191	22.030
	5	0.848	1.113	23.066	21.886
	6	1.184	1.440	23.149	21.978
	7	1.690	2.005	22.660	21.432
	8	2.477	2.986	22.084	20.661
	9	3.695	4.672	20.958	19.067
	10	5.952	7.538	18.745	16.242
10 V${}_{pp}$	1	0.357	0.599	20.036	20.535
	2	0.498	0.755	21.552	21.687
	3	0.714	0.975	22.640	21.797
	4	1.042	1.268	22.643	21.612
	5	1.519	1.793	22.273	21.413
	6	2.204	2.634	21.919	20.582
	7	3.263	3.967	20.866	19.157
	8	5.095	6.509	19.103	16.782
	9	8.516	11.775	15.688	11.532
	10	18.002	N27	6.220	N27
5 V${}_{pp}$	1	0.647	0.883	23.535	22.327
	2	0.941	1.172	22.702	21.555
	3	1.415	1.739	22.767	21.471
	4	2.205	2.710	21.582	20.016
	5	3.374	4.147	20.872	19.178
	6	5.118	6.587	19.138	16.622
	7	8.628	11.804	13.352	9.257
	8	18.109	N27	2.210	N27
	9	—	—	—	—
	10	—	—	—	—

IN27: 2.7V no reached in *t_SCT_* = 20 ms. —: Never turn on.

**Table 11:** Network branch optimized charging times for given input voltages and maximum measurement times using the proposed charging method.

$V_{\mathrm{in}}$	*n*	$t_{a_{n}}$	$t_{d_{stable}}$	$t_{a_{n}}^{*1}$	$t_{d_{stable}}^{*1}$
24	10	15.799	3.763	—	—
18	9	13.696	3.729	15.486	3.780
10	7	10.905	3.282	12.2	3.291
5	5	10.777	3.325	15.5	3.210

*^1^: Extra sensor node reaching *V_limCL_*.
